# Supplementary material for: Assessing the influence of climate on wintertime SARS-CoV-2 outbreaks
Source: Nat Commun. 2021 Feb 8;12:846. doi: 10.1038/s41467-021-20991-1 (PMC7870658; doi:10.1038/s41467-021-20991-1)
Supplement: Supplementary file 1 — Supplementary Information [file 41467_2021_20991_MOESM1_ESM.pdf]

Supplementary information: Assessing the influence of climate on wintertime SARS-CoV-2 outbreaks

Baker et al.

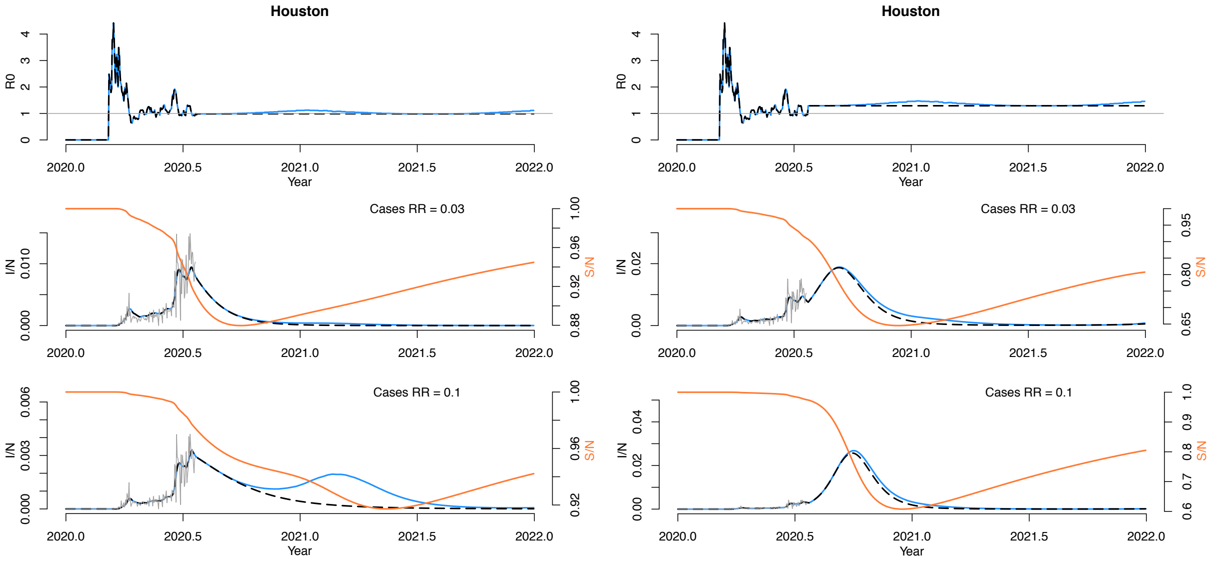

Supplementary Figure 1: **Scenarios for winter outbreaks in Houston**. Left and right column assumes a relative R_0 reduction due to NPIs of 35% and 15% respectively. Middle row and lower row are scenarios assuming a 3% and 10% reporting rate. Cases are show in grey. Proportion susceptible (S) is shown in orange and proportion infected (infected = I/ population = N) is shown in blue.

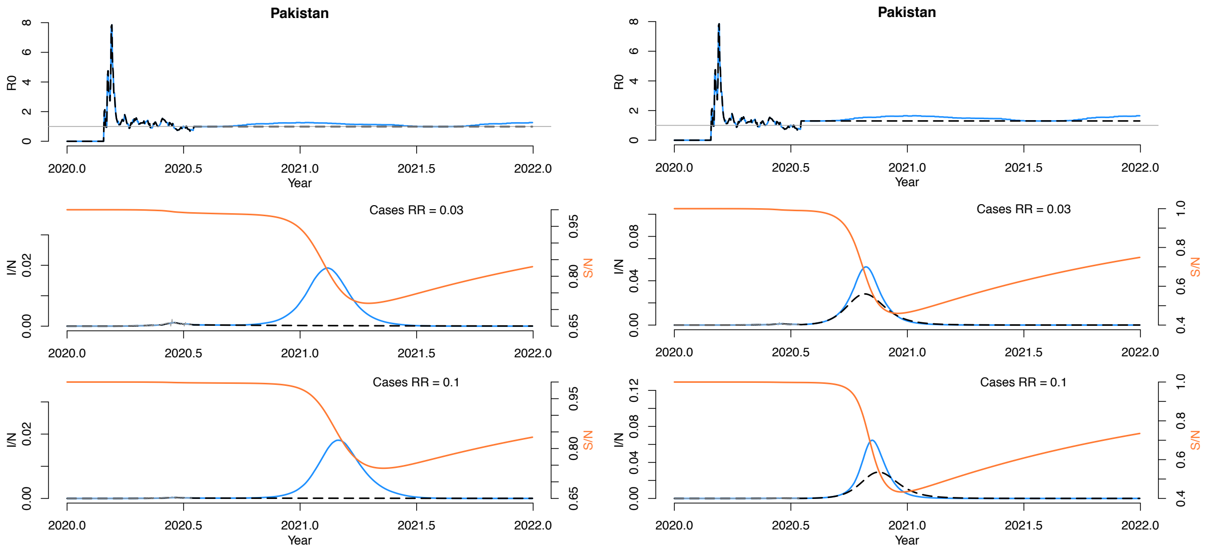

Supplementary Figure 2: **Scenarios for winter outbreaks in Pakistan**. Left and right column assumes a relative R_0 reduction due to NPIs of 35% and 15% respectively. Middle row and lower row are scenarios assuming a 3% and 10% reporting rate. Cases are show in grey. Proportion susceptible (S) is shown in orange and proportion infected (infected = I/ population = N) is shown in blue.

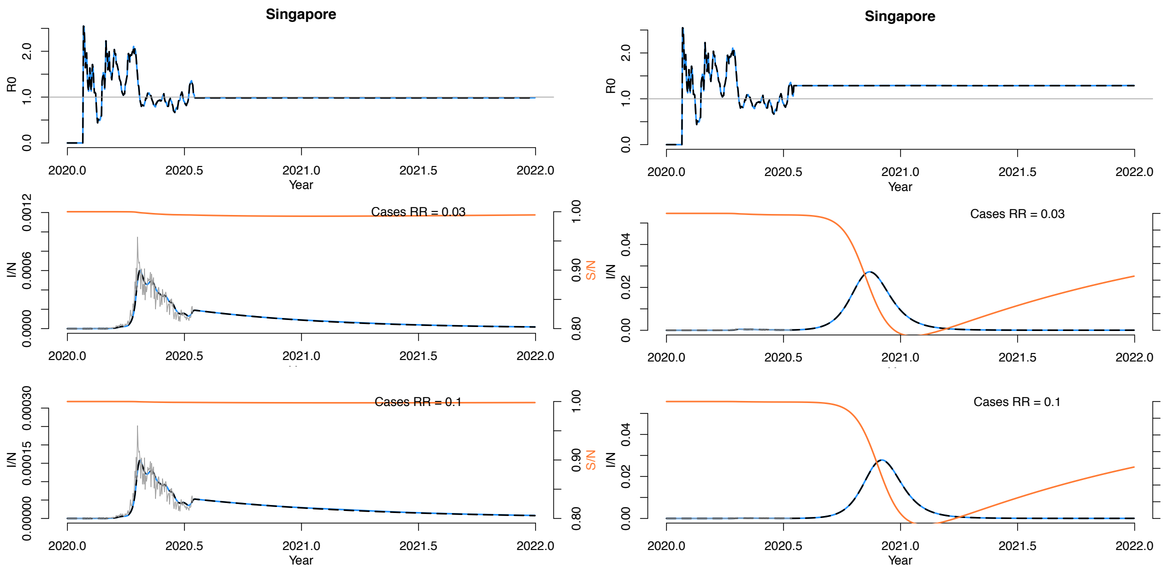

Supplementary Figure 3: **Scenarios for winter outbreaks in Singapore**. Left and right column assumes a relative R_0 reduction due to NPIs of 35% and 15% respectively. Middle row and lower row are scenarios assuming a 3% and 10% reporting rate. Cases are show in grey. Proportion susceptible (S) is shown in orange and proportion infected (infected = I/ population = N) is shown in blue.

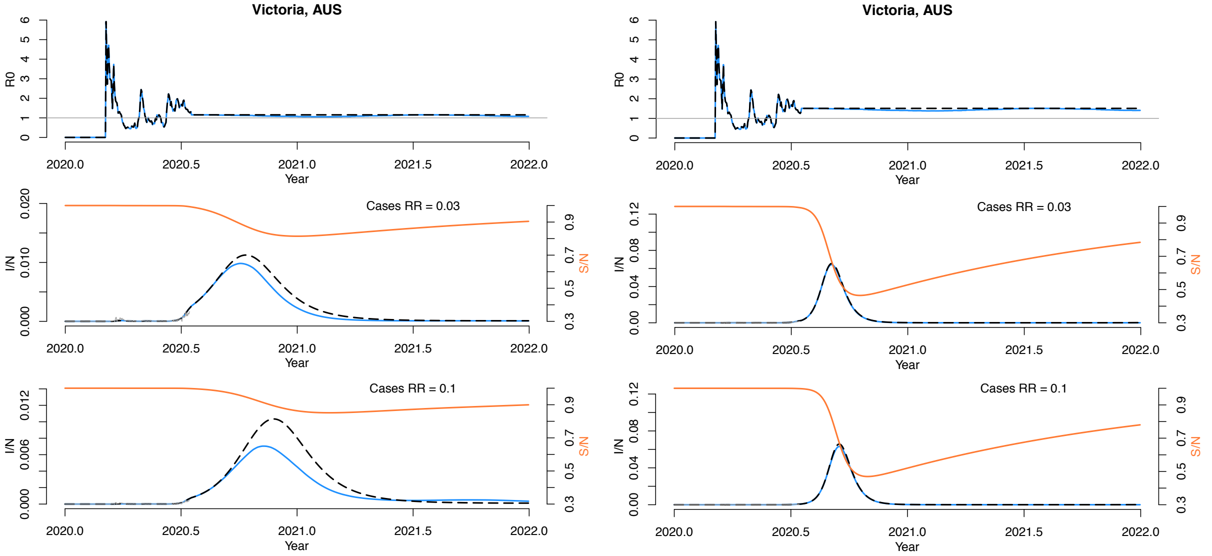

Supplementary Figure 4: **Scenarios for outbreaks in Victoria, Australia**. Left and right column assumes a relative R_0 reduction due to NPIs of 35% and 15% respectively. Middle row and lower row are scenarios assuming a 3% and 10% reporting rate. Cases are show in grey. Proportion susceptible (S) is shown in orange and proportion infected (infected = I/ population = N) is shown in blue. Projections here show much larger case numbers than observed in the region post-July. A second lockdown, enacted in Australia in August 2020, substantially curbed the outbreak.

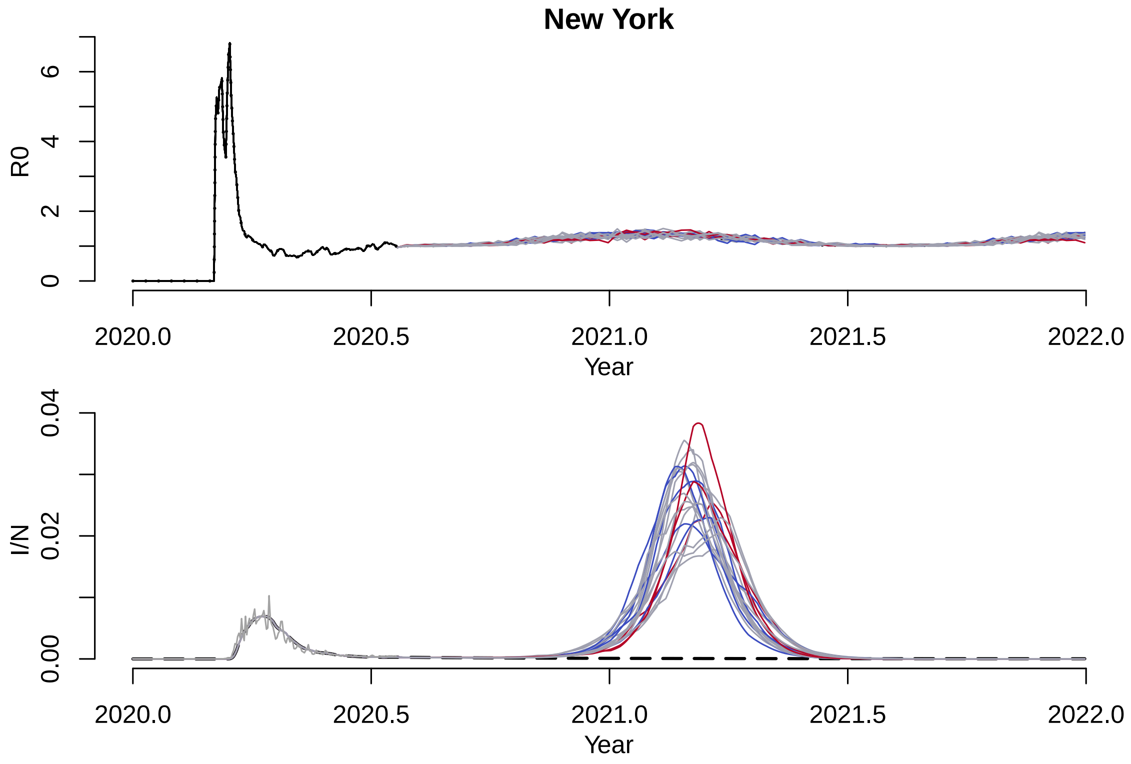

Supplementary Figure 5: **Influence of the north atlantic oscillation on New York outbreaks**. Outbreak simulations are run for New York using the prior 20 years of climate data (assuming a relative reduction in R_0 of 35% and 3% reporting rate). Proportion infected (infected = I/ population = N) for each simulation are shaded by the NAO index where red is > 0.5, blue is < -0.5 and grey is in between. Positive indexes correspond to slightly later peaks, and negative to earlier, however, the effect is not significant.

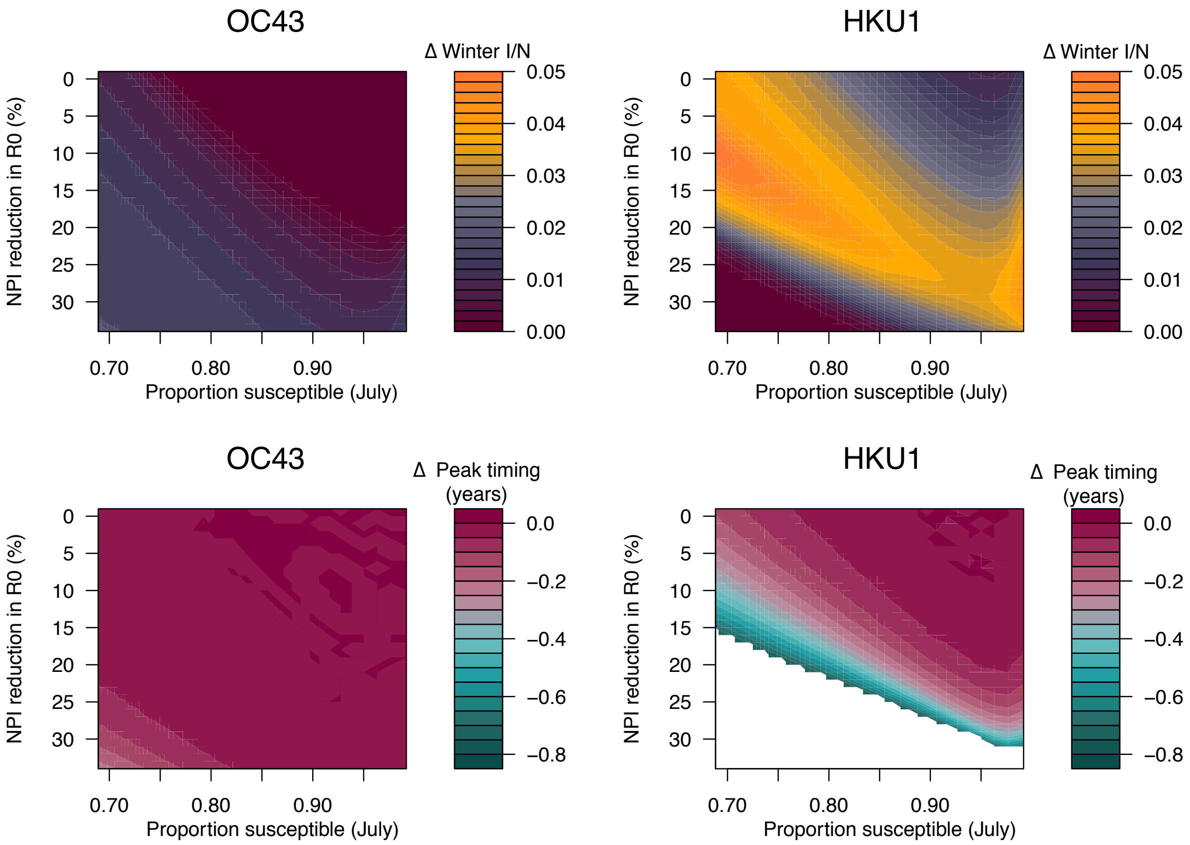

Supplementary Figure 6: **Impact of climate on peak size and timing, HKU1 and OC43 parameters**. Fig. 1 e,h recreated using the climate sensitivity of OC43 (Fig. 1e,h, using HKU1 params are reproduced here for comparison). I = infected and N = population.

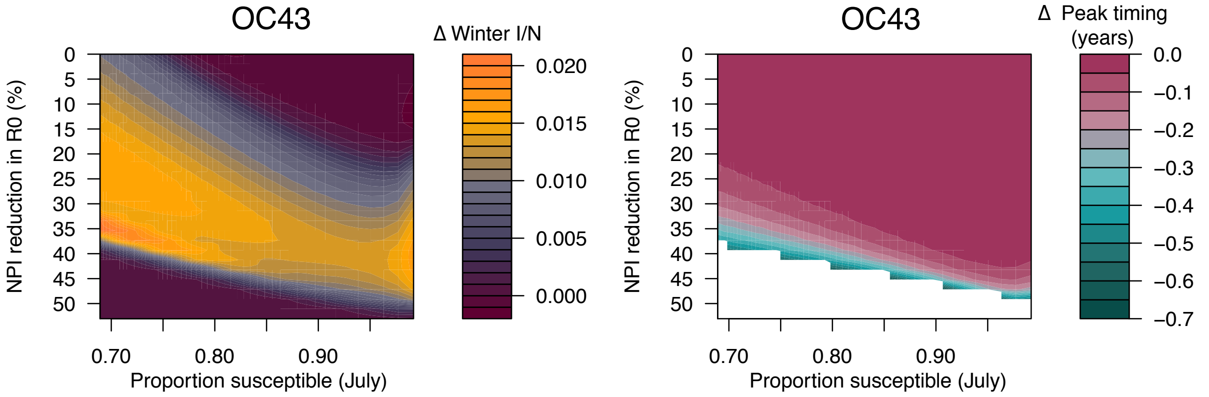

Supplementary Figure 7: **Impact of climate on peak size and timing using expanded range of NPI efficacy (0-55%), OC43 parameters**. The effect of varying the proportion susceptible in July and NPI reduction in R_0 on the relative size of the wintertime peak proportion infected (infected = I/ population = N) in the climate versus constant scenario. The color scale is rescaled to match the maximum $\Delta I/N$ i.e. the color scale does not match Fig. 5 and Fig 1e,h. The plot reveals a similar pattern to Fig. 1e,h, albeit with smaller maximum effect size.

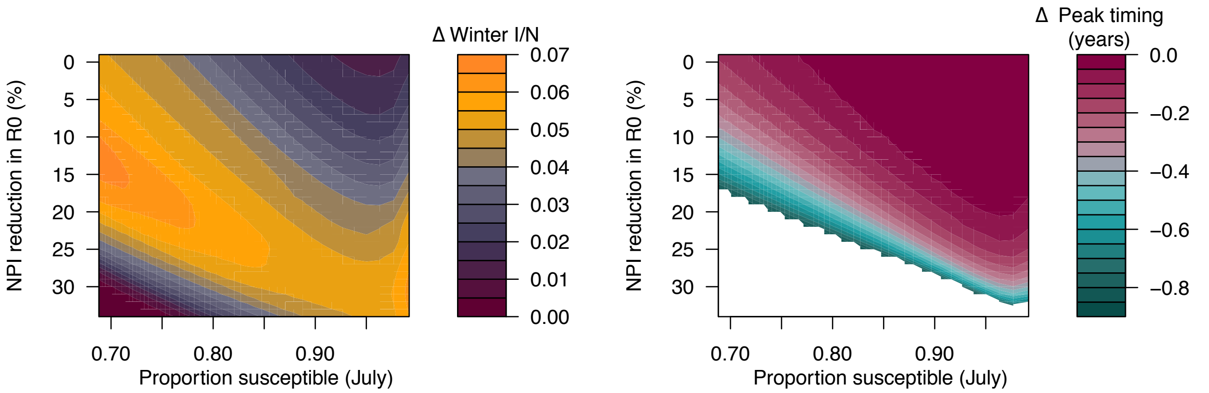

Supplementary Figure 8: **Impact of climate on peak size and timing with larger R_0 range**. Fig. 1 e,h recreated using $R_{0max} = 3$ and $R_{0min} = 1.5$. Climate sensitivity is based on HKU1. I/N is the proportion infected (I = infected/N = population)

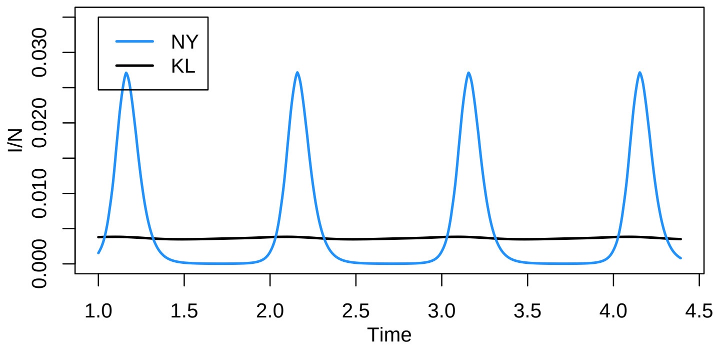

Supplementary Figure 9: **HKU1 model simulations for Kuala Lumpa and New York**. The model is run for 25 years to remove transient dynamics. Results present seasonal fluctuations in the proportion infected (infected = I/ population = N) for the endemic HKU1 infection.

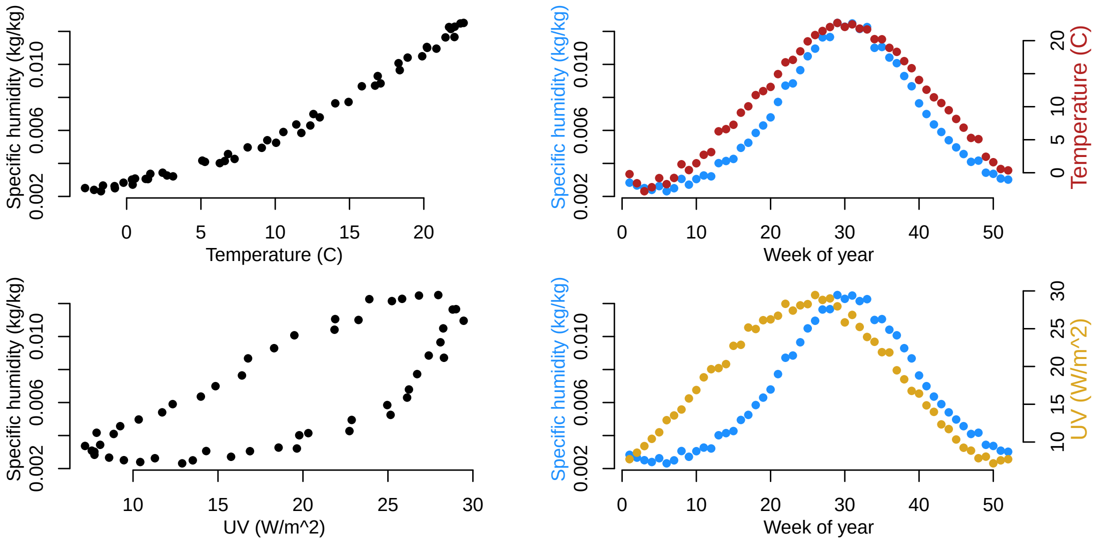

Supplementary Figure 10: **Correlation between specific humidity and temperature/UV**. 30 year average specific humidity, temperature and ultra-violet radiation (UV) for New York City. Temperature and specific humidity are highly correlated (98% correlation coefficient). UV and specific humidity show a lagged relationship (75% correlation).
